# Seed or soil: Tracing back the plant mycobiota primary sources

**DOI:** 10.1111/1758-2229.13301

**Published:** 2024-06-23

**Authors:** Liam Laurent‐Webb, Kenji Maurice, Benoît Perez‐Lamarque, Amélia Bourceret, Marc Ducousso, Marc‐André Selosse

**Affiliations:** ^1^ Institut de Systématique Evolution Biodiversité (ISYEB), Muséum national d'Histoire naturelle, CNRS Sorbonne Université, EPHE Paris France; ^2^ CIRAD, UMR082 LSTM Montpellier Cedex 5 France; ^3^ Institut de Biologie de l'École Normale Supérieure (IBENS), École normale supérieure, CNRS, INSERM Université PSL Paris France; ^4^ Faculty of Biology University of Gdansk Gdansk Poland; ^5^ Institut Universitaire de France Paris France

## Abstract

Plants host diverse communities of fungi (the mycobiota), playing crucial roles in their development. The assembly processes of the mycobiota, however, remain poorly understood, in particular, whether it is transmitted by parents through the seeds (vertical transmission) or recruited in the environment (horizontal transmission). Here we attempt to quantify the relative contributions of horizontal and vertical transmission in the mycobiota assembly of a desert shrub, *Haloxylon salicornicum*, by comparing the mycobiota of in situ bulk soil and seeds to that of (i) in situ adult individuals and (ii) in vitro‐germinated seedlings in soil collected in situ. We show that the mycobiota are partially vertically transmitted through the seeds to seedlings, whereas bulk soil has a limited contribution to the seedling's mycobiota. In adults, root and bulk soil mycobiota tend to resemble each other, suggesting a compositional turnover in plant mycobiota during plant development due to horizontal transmission. Thus, the mycobiota are transmitted both horizontally and vertically depending on the plant tissue and developmental stage. Understanding the respective contribution of these transmission pathways to the plant mycobiota is fundamental to deciphering potential coevolutionary processes between plants and fungi. Our findings particularly emphasize the importance of vertical transmission in desert ecosystems.

## Graphical abstract

The plant microbiota is essential to plant functioning, but the assembly process shaping it is poorly understood, in particular for fungal partners. Here, by coupling in situ sampling and in vitro experiment, we show that the fungal microbiota of *Haloxylon salicornicum*, a desert shrub, is transmitted both vertically through seeds and horizontally through soil depending on the plant compartment and the developmental stage, highlighting the importance of both transmission paths.

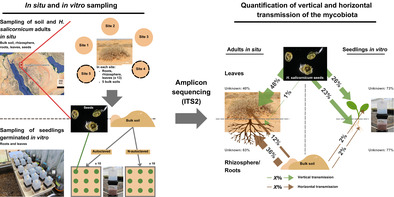

## INTRODUCTION

All plants are colonized on their surface and in their tissues by diverse communities of microorganisms, such as bacteria (Bulgarelli et al., 2013) and fungi (Rodriguez Jr et al., 2009; Selosse et al., 2004). Collectively, these microorganisms are referred to as the microbiota (Berg et al., 2020) for which plants tend to have developed functional dependency (Selosse et al., 2014). Among the plant microbiota, fungi form the mycobiota which play key roles in the plant life cycle as they interact with their plant host in various manners, ranging from parasitic to mutualistic (Trivedi et al., 2020). Though fungi are well‐known to have deleterious effects on plant fitness (e.g., pathogenic fungi), it has been recognized over the past decades that they are also involved in many crucial plant functions such as nutrition (Smith & Read, 2009; Yakti et al., 2018) or defence against pathogens (Rodriguez Estrada et al., 2012). Some fungi also improve plant tolerance to various stresses such as drought (Hosseyni Moghaddam et al., 2021; Li et al., 2018) and soil salinity (Dastogeer et al., 2020; Gonzalez Mateu et al., 2020; Gupta et al., 2021). As a result, plant‐associated fungi increase plants' ecological success (Selosse et al., 2014). To achieve those roles, some fungi form specialized structures with their plant host such as mycorrhizae (van der Heijden et al., 2015), but others colonize their hosts with no apparent symptoms, such as endophytic fungi (sensu Wilson, 1995). Plants and their microbiota are sometimes referred to as ‘holobionts’ (e.g., Vandenkoornhuyse et al., 2015), a theoretical framework stipulating that the accumulation of the plant organism and its microbiota (holobiont) form a unit of selection. This notion is still subject to debate as it implies fidelity between the partners; yet, mutualistic associations tend to be generalist and in most cases are not vertically transmitted across generations (Bright & Bulgheresi, 2010; Douglas & Werren, 2016).

Partners' fidelity may be guaranteed by vertical transmission of the mycobiota (Wilkinson, 1997), that is, if the plant‐associated fungi are transmitted from generation to generation by way of seeds (Bright & Bulgheresi, 2010) and/or during vegetative multiplication (Vannier et al., 2018). Thanks to novel DNA sequencing and barcoding technologies, healthy seeds are no longer considered sterile, as they generally present a diverse and dynamic microbiota at their different developmental stages (Abdelfattah, Tack, Lobato, et al., 2022; Abdelfattah, Tack, Wasserman, et al., 2022; Klaedtke et al., 2016; Nelson, 2018; Simonin et al., 2022), so that fungal vertical transmission through the seeds is possible. However, the sources and mechanisms of acquisition of plant‐associated fungi are still poorly understood. Recent findings suggest that fungi may be acquired by the plant (i) directly from their environment (horizontal transmission, from the soil for instance; Bonito et al., 2014) or (ii) from their parents (vertical transmission) through the seeds (Gundel et al., 2011; Shade et al., 2017) or clonal structures (e.g., aerial stolons; Vannier et al., 2018). Most fungi colonizing belowground tissues (rhizosphere, roots) are considered to be mainly horizontally transmitted from the soil (Bonito et al., 2014; Lundberg et al., 2012). However, vertical transmission was also confirmed in vitro demonstrating that both bacterial and fungal microbiota may be partially transmitted from seeds to seedlings, sometimes over several plant generations (Hardoim et al., 2012; Rezki et al., 2018; Rodríguez et al., 2020). Available knowledge therefore suggests that seedling's mycobiota originate from both seeds and soil in proportions that vary between aerial and belowground tissues. The respective contribution of vertical and horizontal transmission to the microbiota is; however, often considered separately. Yet, taking both pathways into account is crucial to detect potential ubiquitous fungi that could be transmitted both from seeds and soil and thus bias estimations of the contribution of one pathway studied alone. Furthermore, changes in mycobiota composition during plant development (Gao et al., 2019; Houlden et al., 2008) may reflect changes in patterns of horizontal and vertical transmission. To date, only a few studies have comparatively quantified the vertical and horizontal transmission of the microbiota (see Moroenyane et al., 2021; Walsh et al., 2021 for bacteria), especially in fungi (but see Rochefort et al., 2021). The latter study highlights that soil is the main source of seedling mycobiota in *Brassica napus* but only considers early developmental stages. Moroenyane et al. (2021), Rochefort et al. (2021), and Walsh et al. (2021) only used in vitro experimental designs, which may not reflect in situ conditions (e.g., complex plant communities, differences in microbiota composition between substrates used in vitro and soil in situ…). Conversely, samplings only performed in vivo cannot distinguish horizontal from vertical transmission (Perez‐Lamarque et al., 2023). Thus, to the best of our knowledge, no studies have compared vertical and horizontal transmission of the mycobiota at different development stages.

Desert ecosystems represent one third of the world's land surface (Prăvălie, 2016) and their area is expected to increase under climate change (Intergovernmental Panel on Climate Change, 2022). They are characterized by low precipitations and nutrient availability resulting in lower fungal (Tedersoo et al., 2014) and plant (Cai et al., 2023) diversity compared with other biomes. In hot deserts, vegetation is often discontinuous and patchy, with perennial species regularly spaced and separated by bare soil (de Graaff et al., 2014). This patchy distribution of shrubs is referred to as ‘fertility islands’ or ‘resource islands’: soil nutrient concentrations (i.e., C, N, P) close to shrubs are higher than in surrounding bare soils (Schlesinger & Pilmanis, 1998). These fertility islands are therefore hotspots of microbial diversity, including fungi (Maurice, Laurent‐Webb, et al., 2023; Ochoa‐Hueso et al., 2018). The patchy distribution of shrubs may limit the impact of other neighbouring plant species on their mycobiota and in particular the influence of conspecific individuals (Brigham et al., 2023; Schneider‐Maunoury et al., 2020). As they display rather simple and spatially structured plant communities, desert ecosystems are interesting models for in situ ecological research. Furthermore, harsh environmental conditions may limit microbial availability in soil (Maldonado et al., 2022) and we could consequently expect that vertical transmission is favoured. However, decreased vertical transmission has been observed in the leaf's endophytes *Epichloë* spp. under drought conditions (Cavazos et al., 2018). Untangle vertical and horizontal transmission pathways in such constrained environments will therefore provide insights on the mycobiota assembly strategies under stress conditions such as drought.

Here, we take advantage of a desert ecosystem to quantify both vertical and horizontal transmission pathways during mycobiota assembly. We hypothesize that (i) plant mycobiota are mainly influenced by the soil mycobiota with a low contribution of seeds, even at early development stages, and that (ii) different compartments (especially aerial and underground ones) show contrasted patterns of colonization from seeds and soil. We studied *Haloxylon salicornicum* (Amaranthaceae) as a model, a common desert shrub of the Eastern Arabian flora (Al Salameen et al., 2018) with potential for restoration and protection of arid lands (Rathore et al., 2015). We comprehensively assessed the contribution of both horizontal (soil) and vertical (seeds) transmission in the mycobiota assembly of *H*. *salicornicum* through the comparison of mycobiota compositions obtained (i) by sampling in situ bulk soil and the different compartments of adult individuals (rhizosphere, roots, leaves, and seeds) and (ii) by germinating seeds in vitro in non‐processed or autoclaved bulk soil collected in situ. This is, to our knowledge, the first study to date comparing vertical and horizontal transmission at several developmental stages, and the first time that mycobiota assembly strategies are investigated in a desert plant.

## EXPERIMENTAL PROCEDURES

### *In situ sampling of bulk soil, rhizosphere, roots, leaves, and seeds from* H. salicornicum *adult individuals*

**FIGURE 1:**
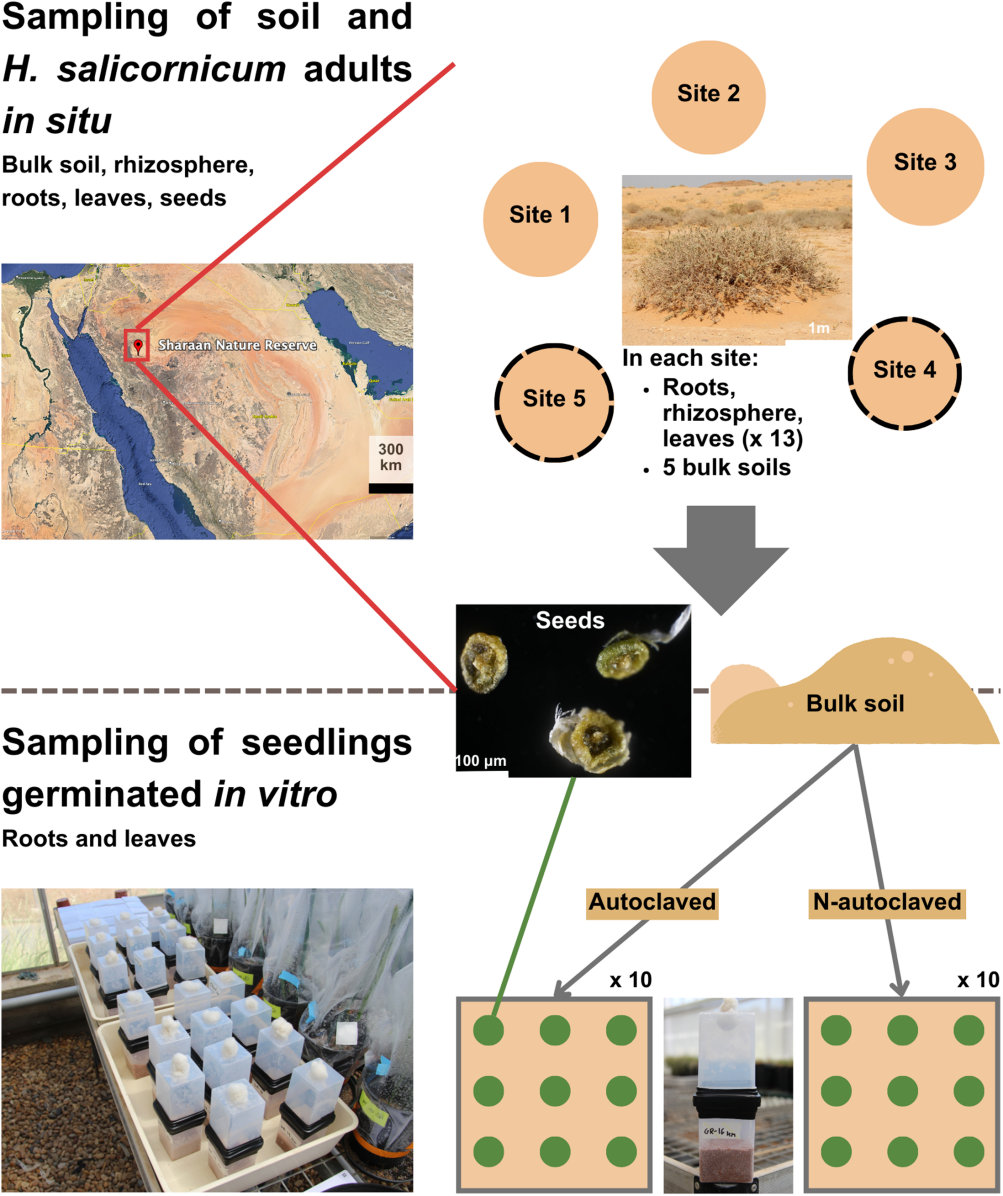
In situ sampling of soil and *H*. *salicornicum* adults' compartments and in vitro seedlings germination experiment. To characterize the mycobiota of adults *H*. *salicornicum*, bulk soil (5 samples per site) along with rhizosphere, roots, leaves, and seeds were sampled from 13 individuals in each of the 5 sites located in the Sharaan Nature Reserve, AlUla Province, Saudi Arabia. Seeds were collected in the study sites during spring 2022. Additional bulk soil samples in sites no. 4 and no. 5 were collected to set up an in vitro germination experiment. In a heated green house, *H*. *salicornicum* seeds were planted in soil collected in situ that was either autoclaved or not (10 pots with 9 seeds in each substrate condition). At two‐leaves stage, roots and leaves of seedlings that successfully germinated were sampled.

Five sites, representative of the different type of soils in which *H*. *salicornicum* flourishes in the Shaaran Natural Reserve, were selected for in situ sampling of bulk soil and *H*. *salicornicum* adult individuals (Province of Medina, AlUla, Saudi Arabia; Figure 1 and Table S1). Soil in these sites ranged from sandy soils with no clay to sandy soils with indurations and higher clay content (Maurice et al., 2023). Soil properties of each site were measured from five bulk soil samples per site collected in March 2022 and are reported in Table S1. All sites are characterized by alkaline soils (pH H_2_O ranges from 8.58 ± 0.58 to 9.14 ± 0.17), low organic matter (0.46% ± 0.05% to 0.7% ± 0.12% of dried soil), low total nitrogen (2 × 10^−2^% ± 3 × 10^−3^% to 3 × 10^−2^% ± 3 × 10^−3^%) and low soil humidity (0.18 ± 0.08 to 2.06 ± 0.8 2% of humidity; Table S2). In March 2022, 5 bulk soil samples were collected along with rhizosphere and roots from 13 individuals in each site for molecular analysis only (Table 1 and Figure 1). Soil samples (bulk and rhizosphere) were sieved to 2 mm and root samples were stored in a 2% cetyltrimethylammonium bromide (CTAB) solution. All samples were stored at 4°C until molecular analyses. In May 2022, we additionally collected phyllosphere from the same individuals than in March 2022 for molecular analysis. No rain events occurred between those two sampling times. As *H*. *salicornicum* harbours small reduced leaves on green photosynthetic stems (Singh et al., 2015), we sampled both stems and leaves together and will refer to them as *leaves*. Sampled leaves were dried using silica gel and kept dry until molecular analyses. As leaves were not sterilized, we studied both epiphytic and endophytic leaf mycobiota. In May 2022, we also collected additional bulk soil from sites no. 4 and no. 5 (which showed no apparent influence of human activities) to use it as substrate for the in vitro experiment. Three bulk soil samples per site (no. 4 and no. 5) were thus collected and all samples were pooled together. Bulk soil samples collected for the in vitro experiment were completely dry at sampling time. They were unprocessed and kept dry for 1 week before setting up the in vitro experiment (see after). Finally, seeds were collected before contact with soil during spring 2022 on *H*. *salicornicum* individuals from the Sharaan Natural Reserve. We randomly sampled 20 pools of 9 seeds from this seed collection for molecular analysis and randomly sampled ca. 200 additional seeds for the in vitro germination experiment. Seeds used for molecular analysis and the in vitro experiment are thus representative of *H*. *salicornicum* seeds' mycobiota in the Sharaan Natural Reserve.

**TABLE 1:** Soil and *H*. *salicornicum* compartments samples collected for mycobiota sequencing.

		Bulk soil	Rhizosphere	Roots	Leaves	Seeds
Collected samples	In situ	25	65	65	65	20 (pools of 9 seeds)
	In vitro	‐	‐	20 (13 + 7)	20 (13 + 7)	‐

*Note*: We sampled bulk soil along with rhizosphere, roots, leaves, and seeds from adult *H*. *salicornicum* individuals in situ to characterize their mycobiota. In vitro, we germinated *H*. *salicornicum* seeds in soil collected in situ that was either autoclaved or not. At the two‐leaves stage, we sampled roots and leaves of seedlings successfully germinated in vitro. The left number in brackets is the number of samples in the autoclaved condition, and the right number (underlined) is the number of samples in the non‐autoclaved condition. Differences in germination rate were not significant (*χ* ^2^ test: *p* = 0.24).

### In vitro experiment

To quantify the contribution of seeds and soil to seedlings' mycobiota, we germinated *H*. *salicornicum* seeds in a heated glass house with a mean temperature of 25–28°C, low humidity, and day/night alternations of 16 and 8 h, respectively. We used unprocessed bulk soil collected in situ as substrate (see above for soil characteristics; Figure 1). Bulk soil was either autoclaved (30 min at 120°C) or not, leading to the two conditions of the in vitro experiment, that is, autoclaved and non‐autoclaved. We expect the autoclaved condition to eliminate most of the fungi present in bulk soil (although some might resist; Wolf et al., 1989). For each condition, 10 Magenta boxes (Merck, Germany) were filled in sterile condition with 250 g of bulk soil as substrate. Before planting, we removed petals and sepals' fragments from seeds with sterile tweezers. Nine seeds per Magenta box were planted at equal distance using sterile tweezers (Figure 1). Substrate was irrigated with sterile Milli‐Q water (40 mL/box). Boxes were closed with another Magenta box, with a 1 cm diameter hole filled with cotton to allow gas exchange and limit aerial contaminants from the glass house. Of the 180 seeds initially planted, 13 reached the two‐leaf stage in the autoclaved condition and 7 in the non‐autoclaved condition (Table 1; differences in germination rate were not significant, χ^2^ test: *p* = 0.24). As the germination rate was low and because we experienced some damping‐off at a later stage during previous trials (data not shown), leaves and roots of seedlings were collected after 7 days (two‐leaf growth stage) under sterile conditions. Neither roots nor leaves showed signs of infection or disease. Roots were washed with sterile water and all were flash‐frozen with liquid nitrogen. Samples were kept at −20°C until molecular analyses.

### Molecular analyses

Bulk soil, rhizosphere, and roots collected in situ were processed as in (Maurice et al., 2023). Leaves and seeds (in situ) and seedling's compartments were processed following the same protocol than roots in Maurice et al. (2023) with slight modifications. Briefly, samples were ground using two sterile stainless‐steel beads in a Tissue Lyzer II (Qiagen, Germany) for 3 × 30 s at 30 Hz. DNA extraction of all samples was performed with the FastDNA Spin Kit for soil (MP Biomedicals™, Solon, USA) following the manufacturer's instructions. DNA concentration of extracts was measured with the PicoGreen fluorophore (Quant‐iT™ PicoGreen™ dsDNA Assay Kits, Thermo Fisher Scientific, USA). DNA concentrations of seedlings' roots were normalized to 0.5 ng/μL^−1^ as in Maurice et al. (2023). Seed and leaf extracts showed higher DNA concentrations (up to 150 ng/μL^−1^; data not shown); we therefore diluted these extracts to 3.5 ng/μL^−1^. The ITS2 region of the fungal ribosomal operon was amplified for all samples with tagged primers ITS86F (5′‐GTGAATCATCGAATCTTTGAA‐3′) and ITS4 (5′‐TCCTCCGCTTATTGATATGC‐3′; Op De Beeck et al., 2014; White et al., 1990). Reactions were performed using the Thermo Scientific Phusion™ High‐Fidelity DNA Polymerase (Thermo Fisher Scientific, USA). All reactions were performed in pseudo‐triplicate. Triplicates were then pooled and checked on 2% agarose gel. PCR products were purified using Agencourt AMPure XP beads (Beckman Coulter Inc., Indianapolis, IN, USA) and their concentrations were measured with PicoGreen. We built two equimolar pools for sequencing: (1) PCR products from in situ samples of bulk soil, rhizosphere, and roots were mixed in one equimolar pool whereas (2) samples of leaves in situ and in vitro, roots in vitro, and seeds were pooled in another equimolar pool. The two pools were purified twice with AMPure XP beads. Each pool was sequenced independently using *MetaFast* library preparation and sequencing (performed by Fasteris SA, Switzerland) on an Illumina platform using the 2 × 250 pb Miseq technology.

### Bioinformatic analysis

A pipeline based on VSEARCH (Rognes et al., 2016) and available on GitHub (https://github.com/BPerezLamarque/Scripts/) was used for data processing (Perez‐Lamarque et al., 2022; Perez‐Lamarque et al., 2022). Briefly, paired‐end reads were merged and quality checked. Merged reads were then demultiplexed using *cutadapt* (Martin, 2011) with 0 error accepted in primer or tag sequences. Reads from all samples were dereplicated and clustered as classical 97% sequence similarity operational taxonomic units (OTUs) using VSEARCH as recommended in Tedersoo et al. (2022). All sequences were checked for the presence of chimeras. The taxonomy of these OTUs was assigned with VSEARCH against the UNITE v9.0 database (Nilsson et al., 2019). Reads were filtered to keep only non‐chimeric sequences of >200 pb and with a total abundance of at least 10 (see Methods S1 for details on the functions and parameters used). We used the *decontam* algorithm (Susana Rivera et al., 2011) to remove potential contaminants, using both *prevalence* and *frequency* algorithms (Methods S1). Samples with <1000 fungal reads were discarded. After filtering, we obtained the mycobiota composition of 259 samples (96% of all collected samples; Table S1), with a mean sequencing depth of 21,013 reads per sample (ranging from 1041 to 130,227; Figures S1 and S2). To compute UniFrac distances, we reconstructed the fungal phylogenetic trees as in Perez‐Lamarque et al. (2022), Perez‐Lamarque et al. (2022; Methods S1).

### Statistical analysis

OTU tables were processed using the *phyloseq* package (McMurdie & Holmes, 2013) in R (R Core Team, 2023).

#### Richness and diversity analysis

We computed the richness (Chao1 estimator) and diversity (Shannon index) of each sample based on actual counts using the *vegan* R package (Oksanen et al., 2013). To test the significance of differences between experimental designs (in situ and in vitro), compartments (bulk soil, rhizosphere, roots, leaves, and seeds), and substrate conditions in vitro (non‐ and autoclaved bulk soil), we used linear regression and Tukey's post hoc pairwise test (Methods S2).

#### Assessing differences in community structure

To test for differences in mycobiota composition between experimental designs, compartments, and substrate conditions, we performed β‐diversity analyses. We computed Bray–Curtis distances using either relative abundances (as they may perform better for community comparisons; Gloor et al., 2017; McKnight et al., 2019) or Hellinger‐transformed data to correct for variability in sampling depth (Legendre & Gallagher, 2001). We also computed UniFrac distances to account for the phylogenetic relatedness between OTUs in our community composition and clustering analyses. Bray–Curtis distances were computed using the *vegan* package (Oksanen et al., 2013) while UniFrac distances were computed using the *phyloseq* package. We visualized community composition differences using principal coordinate analysis (PCoA). As both transformations (relative abundance and Hellinger transformation) and both distances (Bray–Curtis and UniFrac) showed similar results, we only report results from Bray–Curtis distances computed using relative abundances in the main text. The significance of differences in mycobiota composition between the two experimental designs and between compartments was tested using *Permutational Analysis of Variance* (*PERMANOVA*, 10,000 permutations) with all samples using the following model: *distance*~*exp*. design*compartment (where *exp*. *design* corresponds to in situ or in vitro). Then, to test whether the two substrate conditions in vitro led to different mycobiota composition and to test for differences in composition between above‐ and belowground compartments, we used a second *PERMANOVA* (10,000 permutations) with only leaves and roots of individuals germinated in vitro using the following model: *distance*~compartment*substrate condition.

#### Construction of plant/fungal bipartite networks to assess the sharing of OTUs

To further assess the sharing of OTUs between compartments (in particular between bulk soil, seeds, and other plant tissues), we constructed bipartite networks using the *igraph* R package (Csardi & Nepusz, 2006). Indeed, if the mycobiota of a plant compartment (e.g., roots) mainly assemble from bulk soil (horizontal transmission), samples from that compartments should share more fungal OTUs with the soil samples than with the seed samples. We therefore aim at identifying the pairs of compartments that share many fungal OTUs. Bipartite networks are visualized using the Fruchterman–Reingold algorithm (Fruchterman & Reingold, 1991), such that samples sharing many OTUs tend to cluster together. We built one network with all samples and a subnetwork to give a clearer representation of the in vitro experiment (including seedlings, seeds, and bulk soils used for this experiment). To test whether OTUs are shared preferentially between samples from the same compartment or not, we built one network for each compartment of the in situ dataset and one network by compartment x substrate condition combinations in the in vitro experiment. For each network, we consider an interaction with a fungal OTU as long as the latter represents at least 0.5% of the reads to avoid rare interactions that may be spurious. We computed the connectance C (i.e., the number of realized links divided by the number of potential links; function *networklevel*, *bipartite* R package) and the network specialization index H_2_′ (degree of specialization of the entire network; function *H2fun*, *bipartite* R package) of each network (Dormann et al., 2008). Connectance ranges from 0 to 1; values close to 1 indicate that a high proportion of potential links are realized. H_2_′ also ranges from 0 to 1, 0 indicating a strong generalization and 1 that the network is highly specialized. H_2_′ is robust against differences in sampling size and is therefore suited for network comparisons (Blüthgen et al., 2006). Significance of both connectance (C) and specialization (H_2_′) was tested using null models (Methods S2).

#### Source tracking analysis

To estimate the respective contributions of seeds (vertical transmission) and bulk soil (horizontal transmission) to the *H*. *salicornicum* mycobiota, we used the *fast expectation–maximization for microbial source tracking* (FEAST) algorithm developed by Shenhav et al. (2019) and implemented in R. This algorithm estimates the fraction of a microbial community (the ‘sink’) that can be explained by potential microbial sources (the ‘sources’). The algorithm also reports an unexplained fraction referred to as the ‘unknown’ source. Here, seeds and bulk soil samples were defined as ‘sources’ whereas roots, rhizosphere, and leaves were defined as sinks. We ran the procedure twice: first, on the in situ adult individuals' compartments (with in situ seeds and bulk soil as sources) and, a second time on seedlings from the in vitro experiment (with in situ seeds and bulk soil samples from sites used for the germination experiment as sources). To test whether differences in contribution from bulk soil and seeds were significant, we used linear regressions and Tukey's post‐hoc test (Methods S2). As seeds' mycobiota may also originate from other compartments (especially as in situ samples were collected the same year), we also ran the procedure with seeds defined as sink and bulk soil, rhizosphere, and leaves of adults in situ as sources.

#### Identifying potentially transmitted OTUs

We identified OTUs potentially transmitted from the sources (bulk soil and seeds) to the compartments (or sinks), using a ‘strict’ and a ‘loose’ definition. With our strict definition, a potentially transmitted OTU is an OTU shared between one source and one sink (e.g., between bulk soil and rhizosphere), but absent from the other source (seeds in this example). This definition ensures that the OTUs identified as potentially transmitted can only be transmitted from one of the two sources. With our loose definition, potentially transmitted OTUs are OTUs shared between one source and one sink (e.g., bulk soil and rhizosphere) but not necessarily absent from the other source (here seeds). This second definition allows us to identify ubiquitous OTUs that may be transmitted both horizontally and vertically. For each definition, we represented the composition of the fraction of the mycobiota explained by potentially transmitted OTUs, that is, the mycobiota composition when considering only potentially transmitted OTUs. We also computed the mean share of the mycobiota they represent.

## RESULTS

We successfully sequenced the fungal ITS2 region of 259 samples, including roots, rhizosphere, and leaves of in situ *H*. *salicornicum* individuals and their associated bulk soil (i.e., with no apparent vegetation). We also characterized the mycobiota of seeds collected in the same area and leaves and roots of *H*. *salicornicum* seedlings germinated in vitro in non‐ and autoclaved soil (see Table S1 for details on successfully processed samples). The mean sequencing depth for these samples was 21,013 reads (ranging from 1041 to 130,227; Figures S1 and S2) after removing extraction and PCR contaminants. Rarefaction curves tended to reach a plateau for most of the samples (Figure S2), suggesting that our sampling properly encompasses the mycobiota diversity of soil and *H*. *salicornicum* tissues.

### Haloxylon salicornicum *displays contrasted mycobiota diversity between compartments in adults in situ but not in seedlings in vitro*

**FIGURE 2:**
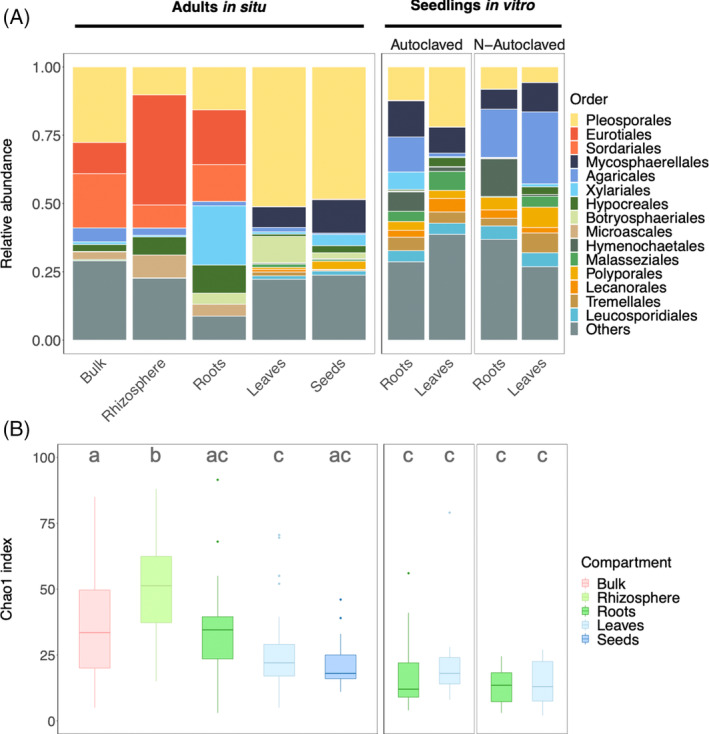
*Haloxylon salicornicum* mycobiota vary according to the compartment in adults in situ but not between compartments or substrate condition in seedlings in vitro. Both composition (A) and richness (B) varied according to the compartment in adults in situ while no differences seemed to occur between compartments and substrate conditions in seedlings in vitro. (A) Mycobiota composition (in relative abundance) of *H*. *salicornicum* compartments in situ and in vitro at the order level. Bar plots represent the mean proportion of each fungal order by compartment, experimental design, and substrate condition in vitro. For in vitro samples, both substrate conditions (non‐ and autoclaved) are shown. For readability purposes, only the main 15 orders are shown, while the rest are aggregated in the ‘Others’ category. See Supp. Figure 3 for mycobiota composition at the genus level. (B) Fungal richness (Chao1 index) in the different compartments, experimental designs (in situ and in vitro), and substrate conditions in vitro. Different letters indicate significant differences (*p* < 0.05; Tukey's post hoc pairwise test).

Adult individuals in situ harboured contrasted mycobiota compositions depending on the studied compartment, in particular between above‐ and belowground compartments, whereas seedlings in vitro displayed similar leaf and root mycobiota in the two substrate conditions (non‐ and autoclaved; Figures 2 and S3).

In situ, soil compartments (bulk soil, rhizosphere, and roots) were characterized by a greater proportion of Eurotiales and Sordariales compared with aerial compartments (leaves and seeds) which were mainly colonized by Pleosporales (representing almost 50% of the mycobiota for both aerial compartments) and to a smaller extent by Mycosphaerellales (Figure 2A; see Figure S3 for mycobiota composition at the genus level). Adult roots were also characterized by a larger share of Xylariales (Figure 2A) compared with other compartments. The share of Botryosphaeriales was higher in leaves than in seeds. In vitro, we observed small differences between seedling roots and leaves and between substrate conditions (Figures 2A and S3). Seedlings in vitro harboured a small share of Pleosporales compared with in situ leaves and seeds but similar proportions of Mycosphaerellales. Contrary to adult roots in situ, roots of seedlings in vitro harboured almost no Eurotiales or Sordariales and a small share of Xylariales. Interestingly, despite similar mycobiota composition at the order level, the top five OTUs were quite different between compartments and substrate conditions in vitro, except for *Mycosphaerella asteroma* which was ubiquitous (File S1).

The highest fungal richness was observed in the rhizosphere of adults in situ (*chao1* = 49 ± 17; Figure 2B), followed by bulk soil and roots in situ (37 ± 22 and 33 ± 15, respectively). Among samples in situ, leaves and seeds harboured the lowest richness (25 ± 13 and 22 ± 11, respectively). All in vitro samples had lower richness compared with samples in situ (ranging from 13 ± 8.1 for roots in the non‐autoclaved condition to 22 ± 18 for leaves in the autoclaved condition; *ANOVA*: *p* < 10^−15^; Table S3). Differences in richness were not significant between substrate conditions in vitro. Similarly, differences in richness between roots and leaves in vitro were not significant (*p* = 0.54, *ANOVA*; Table S3), though leaves tended to harbour a slightly higher richness (Figure 2B and Table S3). The Shannon diversity index revealed similar trends (Figure S3 and Table S4). Diversity and richness therefore displayed similar patterns: in seedlings in vitro, no significant differences between roots and leaves and substrate conditions were identified (Figures S4 and Tables S3 and S4), while in adults in situ, compartments differed significantly in richness and diversity.

### Mycobiota communities are highly structured in two main groups

**FIGURE 3:**
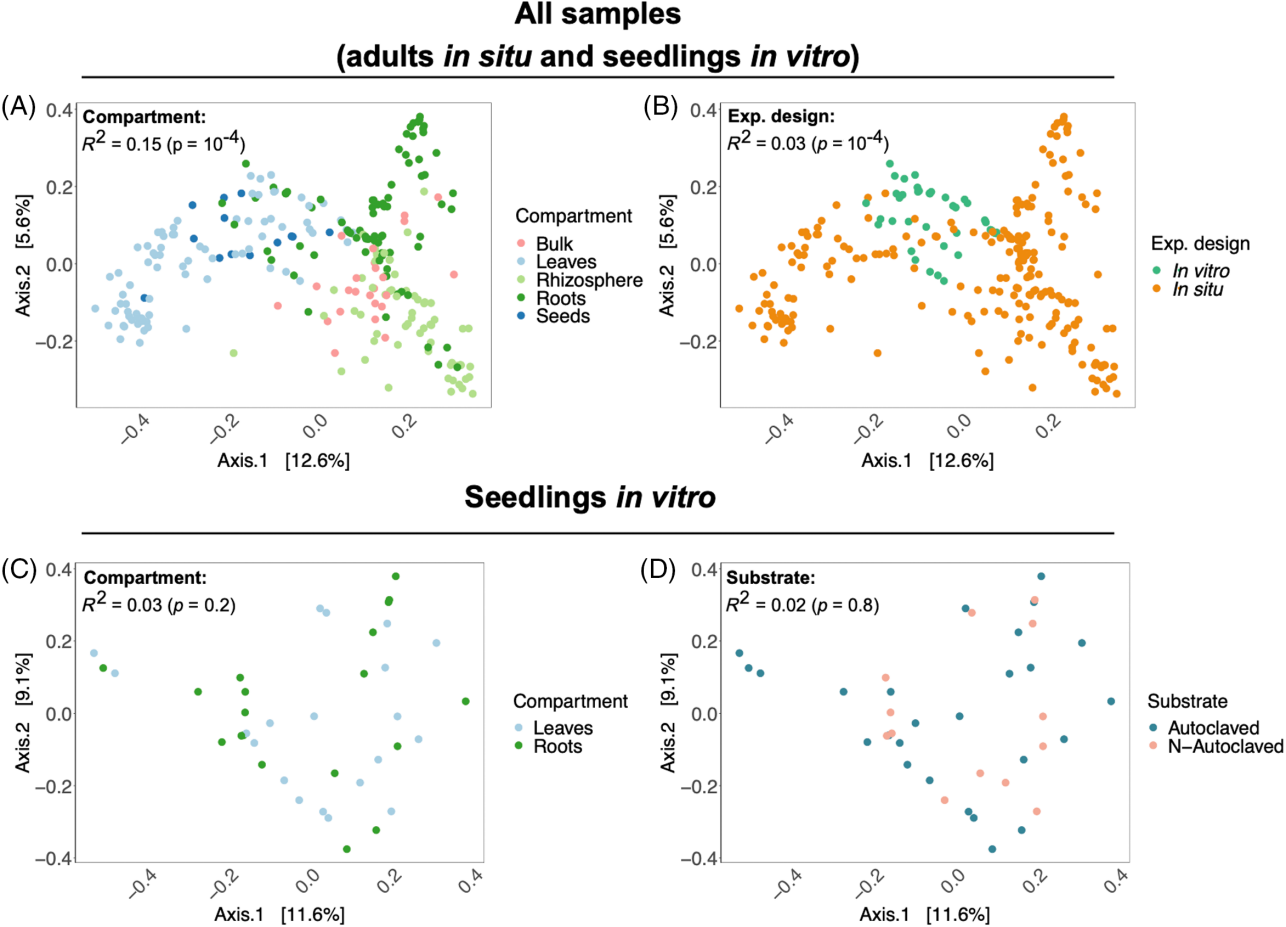
*Haloxylon salicornicum* fungal communities significantly differ between compartments and experimental design (in situ vs. in vitro). Principal coordinate analysis (PCoA) of the mycobiota composition, based on Bray–Curtis distances computed using relative abundances. The influence of the variables on Bray–Curtis distance matrices was tested using *PERMANOVA* (10,000 permutations). (A,B) All samples. (C,D) Seedlings' roots and leaves (in vitro). (A) The compartment explains a large share of the differences in mycobiota composition between all samples. (B) The experimental design also has a significant influence on the mycobiota composition. (C,D) When considering only in vitro samples, neither compartment (C) nor substrate condition (D) has a significant influence on the mycobiota.

**FIGURE 4:**
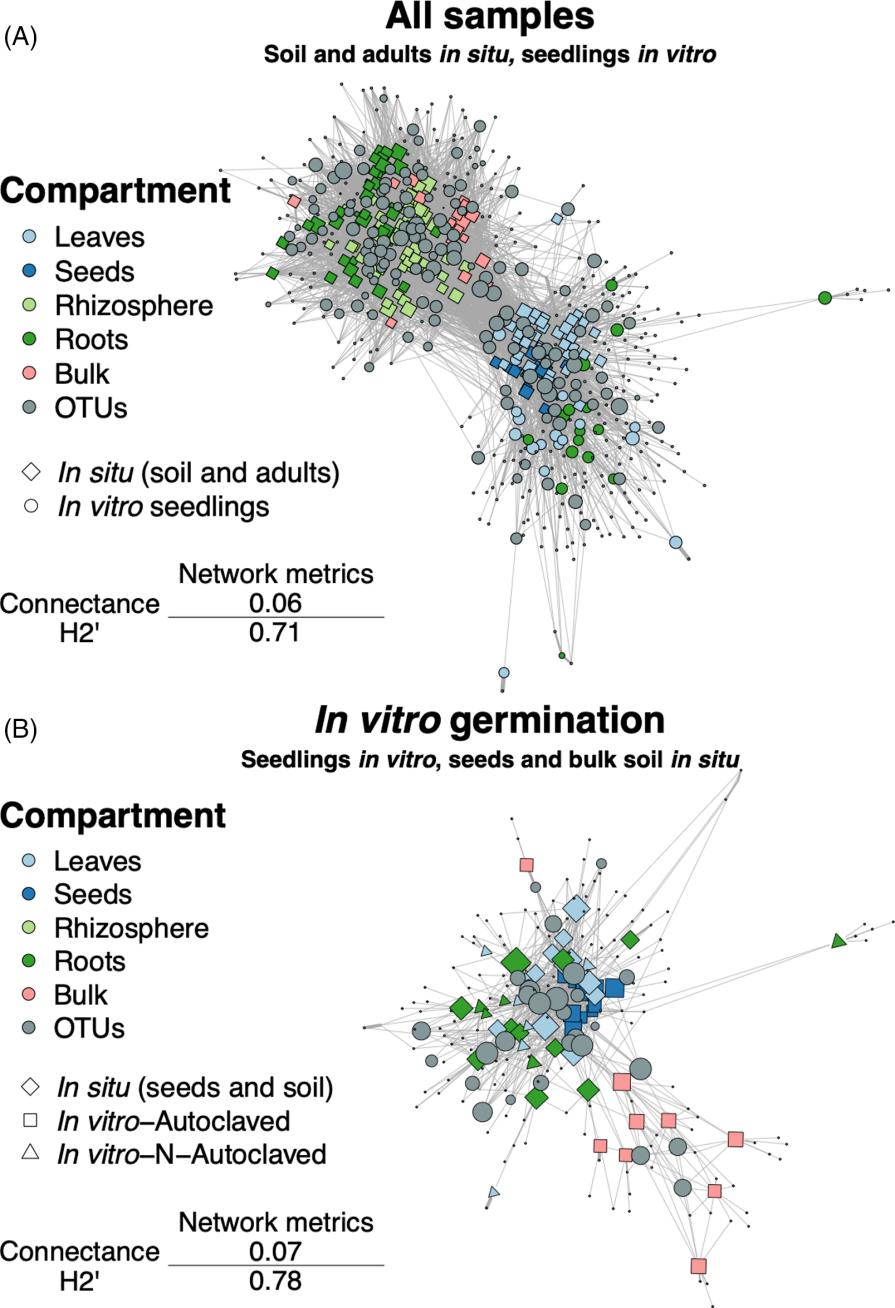
Bipartite networks are structured in two groups preferentially sharing fungal operational taxonomic units (OTUs). (A) Bipartite network of all samples reveals two groups of samples sharing more fungal OTUs: bulk soil, adults' rhizosphere and roots in situ form one group whereas adults' leaves and seeds in situ and seedlings' roots and leaves in vitro form a second one. (B) When focusing on samples of the in vitro germination experiment (i.e., adults' seeds collected in situ, bulk soil used as substrate and seedlings' leaves and roots), we confirm that seedlings' leaves and roots share more OTUs with seeds than with bulk soils used as substrate. No differences between substrate conditions are observed.

As revealed by β‐diversity and bipartite network analyses, two groups of samples tend to have similar mycobiota composition and to share more fungal OTUs, that is: bulk soil, rhizosphere, and roots of adults in situ on the one hand, and leaves, seeds and seedlings' roots and leaves on the other hand (Figures 3 and 4).

As illustrated by the principal coordinate analysis (PCoA; Figure 3), samples from the same compartment tended to cluster together. Differences in mycobiota composition between compartments were significantly distinct (*PERMANOVA*: *R* ^2^ = 0.15, *p* < 10^−4^; Figure 3A). Significant differences between in situ and in vitro fungal communities were also observed (*R* ^2^ = 0.03, *p* < 10^−4^; Figure 3B). Globally, in situ soil compartments tended to cluster together, while in situ aerial compartments and in vitro samples tended to cluster in a second group (Figure 3A,B). We performed the analysis with only in vitro samples to test for differences between compartments and substrate conditions (non‐ and autoclaved soil) and we observed no significant influence of the compartment nor substrate condition (*p* > 0.1 for both variables and their interaction term; Figure 3C,D). These results were consistent when using Hellinger‐transformed data instead of relative abundances and when using UniFrac distances instead of Bray–Curtis distances (Figure S5).

Weighted bipartite networks (based on relative abundances of OTUs) are visualized using the Fruchterman–Reingold layout algorithm, which allow better readability. Grey nodes represent OTUs and coloured ones represent samples. Node diameter is proportional to the betweenness centrality. Width of the edges is proportional to the relative abundance of an OTU in a sample. See Methods S2 for connectance (C) and specialization (H_2_′) calculation and significance tests.

We also constructed two bipartite networks to compare the structure of the fungal communities and assess the sharing of OTUs between compartments, experimental design, and substrate condition. When considering the total network (Figure 4A), we identified two main modules: one with in situ soil compartments (bulk soil, rhizosphere, roots) and another one with samples in vitro, seeds, and leaves in situ. More OTUs were shared within than between the modules, despite some OTUs with high betweenness centrality (i.e., central OTUs that link many nodes of the network) linking them. The total network had a low connectance (C = 0.06), meaning that a low proportion (6%) of all possible interactions between OTUs and samples was observed, which is significantly lower than expected based on our null model (*p* < 0.025). Furthermore, the specialization of the network was significantly higher than expected by chance (H_2_′ = 0.71; *p* < 0.025). Altogether, these results suggest a limited sharing of OTUs between samples. When focusing on the network of the in vitro experiment (formed by seeds and bulk soils used as substrate for the in vitro experiment and seedlings' leaves and roots; Figure 4B), we observe that plant tissues tended to cluster together while bulk soils were more peripheral (Figure 4B). Samples from the non‐ and autoclaved conditions did not seem to form specific groups, a result consistent with the *PERMANOVA* analysis (*R* ^2^ = 0.02, *p* = 0.8; Figure 3C,D). When considering each compartment separately (in each substrate condition for seedlings in vitro), we observed that the connectance of the networks was ca. four times higher compared with the total network (Figure S6), suggesting that the low connectance of the networks in Figure 4 is linked to a low sharing of OTUs between compartments compared with the sharing within compartments. In situ, soil compartment networks tended to be more specialized (bulk: H_2_′ = 0.7; rhizosphere: H_2_′ = 0.61; roots: H_2_′ = 0.75; Figure S6a) compared with aerial compartments (leaves: H_2_′ = 0.46; seeds: H_2_′ = 0.58; Figure S6a) while in vitro, leaves and roots showed similar specialization in both conditions (Figure S6b), except for roots in the non‐autoclaved condition which displayed higher specialization (H_2_′ = 0.91; Figure S6b).

### Soil contributes to adults' root and rhizosphere mycobiota in situ, while seeds contribute to adults' leaf mycobiota in situ and seedlings' leaf and root mycobiota in vitro

**FIGURE 5:**
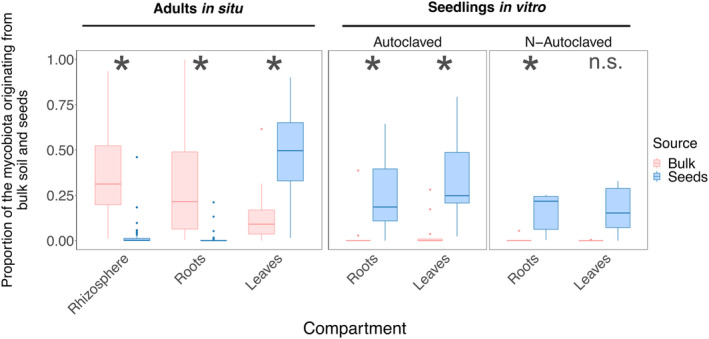
Vertical and horizontal transmission differ between adults in situ and seedlings in vitro and between compartments according to the source tracking analysis. The proportion of the mycobiota originating from bulk soil and seeds varies between compartments and experimental design. Bulk soil is the main source of mycobiota for rhizosphere and roots in situ while seeds explain most of the mycobiota of leaves in situ and of seedlings' leaves and roots in vitro. Proportions were estimated using the *fast expectation–maximization for microbial source tracking* (FEAST) algorithm. Asterisks indicate significant differences between the proportions of the two sources, based on Tukey's post hoc pairwise test (see Methods S2 and Figure S7a–c).

**FIGURE 6:**
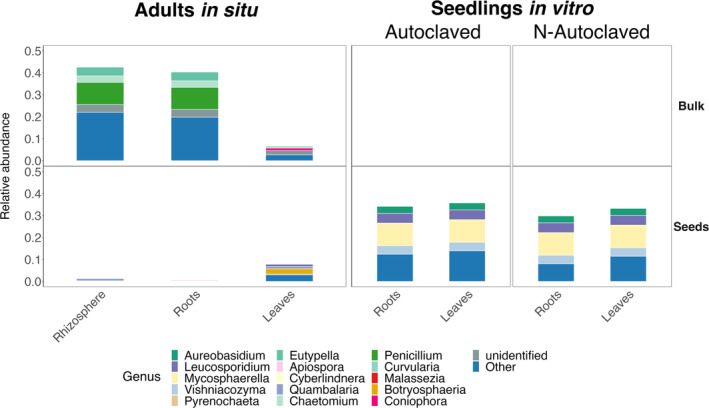
Different fungal genera are potentially transmitted from bulk soil and seeds to *H*. *salicornicum* compartments. We identified potentially transmitted operational taxonomic units (OTUs) from sources (bulk soil and seeds) to *H*. *salicornicum* compartments for adults in situ and seedlings in vitro (in both substrate conditions) using a strict definition: OTUs are considered potentially transmitted if they are shared between a source (e.g., bulk soil) and the studied compartment (e.g., roots) but not with seeds (in this example). We then represented the share of the mycobiota (at the genus level) of each plant compartment when considering only potentially transmitted OTUs with bulk soil or seeds. Figure S7 for similar plots using the loose definition of potentially transmitted OTUs.

The source tracking algorithm *FEAST* and the identification of potentially transmitted OTUs revealed variable contributions of seeds (vertical transmission) and bulk soil (horizontal transmission) as potential fungal sources to *H*. *salicornicum* mycobiota (Figures 5 and 6). In vitro, both leaf and root mycobiota in the two substrate conditions (non‐ and autoclaved) were mainly explained by seed mycobiota (ranging from 16% to 33%; Figure 5 and Table S5), whereas the bulk soil contribution was very low (ranging from 0.1% for leaves in the non‐autoclaved condition to 4.0% for leaves in the autoclaved condition). The contribution of seeds was significantly greater than the contribution of bulk soil in vitro irrespective of the compartment and substrate conditions (*p* > 0.5 for both variables, *ANOVA*; Table S6; Figure S7a for results and Figure S7b,c for regression diagnostic plots) suggesting a predominance of vertical transmission in seedlings in vitro (except for leaves of the non‐autoclaved condition; Figure 5). In contrast, for adults in situ, we observed different transmission pathways: roots and rhizosphere mycobiota originate mainly from bulk soil (mean contribution of respectively 39% and 32%), while seeds contribute significantly less (respectively 2.0% and 0.8%; Figures 5 and Table S5). Conversely, in situ, leaf mycobiota were mostly explained by the seeds (mean contribution of 48%) with a minor contribution from the bulk soil (12%). As seeds were harvested on adults in situ during the same year than other compartments, their mycobiota may also originate from the latter. In particular, *FEAST* analysis with seeds sinks and other compartments of adults in situ as sources show that leaves may highly contribute to seeds' mycobiota (mean contribution of 63%; Figure S8). On the contrary, belowground compartments have a low contribution to seeds' mycobiota (bulk soil: 2.03%; rhizosphere: 2.15%; roots: 0.58%; Figure S8).

We identified fungal candidates for transmission using a strict and loose definition of potentially transmitted OTUs (File S2). Using the strict definition, we confirmed the prevalence of OTUs sharing between seeds and seedlings: between 22 and 59 OTUs were shared between seeds and seedlings in vitro, representing 30%–43% of their mycobiota (Figures 6 and Table S7). In particular, *Mycosphaerella* and *Leucosporidium* OTUs were shared between seeds and roots/leaves in the two substrate conditions (Figure 6). Conversely, seedlings in vitro shared very few OTUs with the bulk soil (Figure 6), representing a null (or close to) contribution of their mycobiota (Table S7). This markedly contrasts with the rhizosphere and roots of adults in situ that shared a high number of OTUs with the bulk soil (respectively, 226 and 159; Figure 6), representing a large share of their mycobiota (43% and 40%, respectively; Table S7). Among candidates for transmission between bulk soil and rhizosphere/roots, we mainly identified OTUs belonging to the *Penicillium*, *Chaetomium*, and *Eutypella* genera (Figure 6). A very small number of OTUs were shared between seeds and rhizosphere/roots in situ, representing a small share of their mycobiota (Table S7). Bulk soil and leaves of adults in situ shared 46 OTUs, which represent 6.8% of the leaf mycobiota. This percentage is similar to the sharing between seeds and leaves in situ (54 OTUs, 7.9% of the mycobiota); this contrasts with the results from the source tracking analysis which indicated that the seeds contributed 48% of the leaf mycobiota in situ (Figure 5 and Table S5). The apparent contradiction between the two methods was overcome when using our loose definition of potentially transmitted OTUs as we observed that some ubiquitous fungi (such as fungi from the genus *Alternaria*; Figure S9) may be transmitted by both seeds and bulk soil to rhizosphere, roots, and leaves in situ. With this loose definition, potentially transmitted OTUs from seeds and bulk soil represented almost 40% of leaves in situ (Figure S9).

Altogether, the source tracking analysis and the identification of potentially transmitted OTUs were consistent, as the number of potentially transmitted OTUs (Figure S10a) and their share of the mycobiota (Figure S10b) were correlated to the proportion of the mycobiota explained by the source tracking analysis. They suggested a predominance of vertical transmission from seeds to seedlings in vitro (regardless of the compartment) and a predominance of horizontal transmission in soil compartments of adults in situ. Leaves of adults in situ displayed a mixed pattern, with a predominance of vertical transmission but a significant share of horizontal transmission.

## DISCUSSION

**FIGURE 7:**
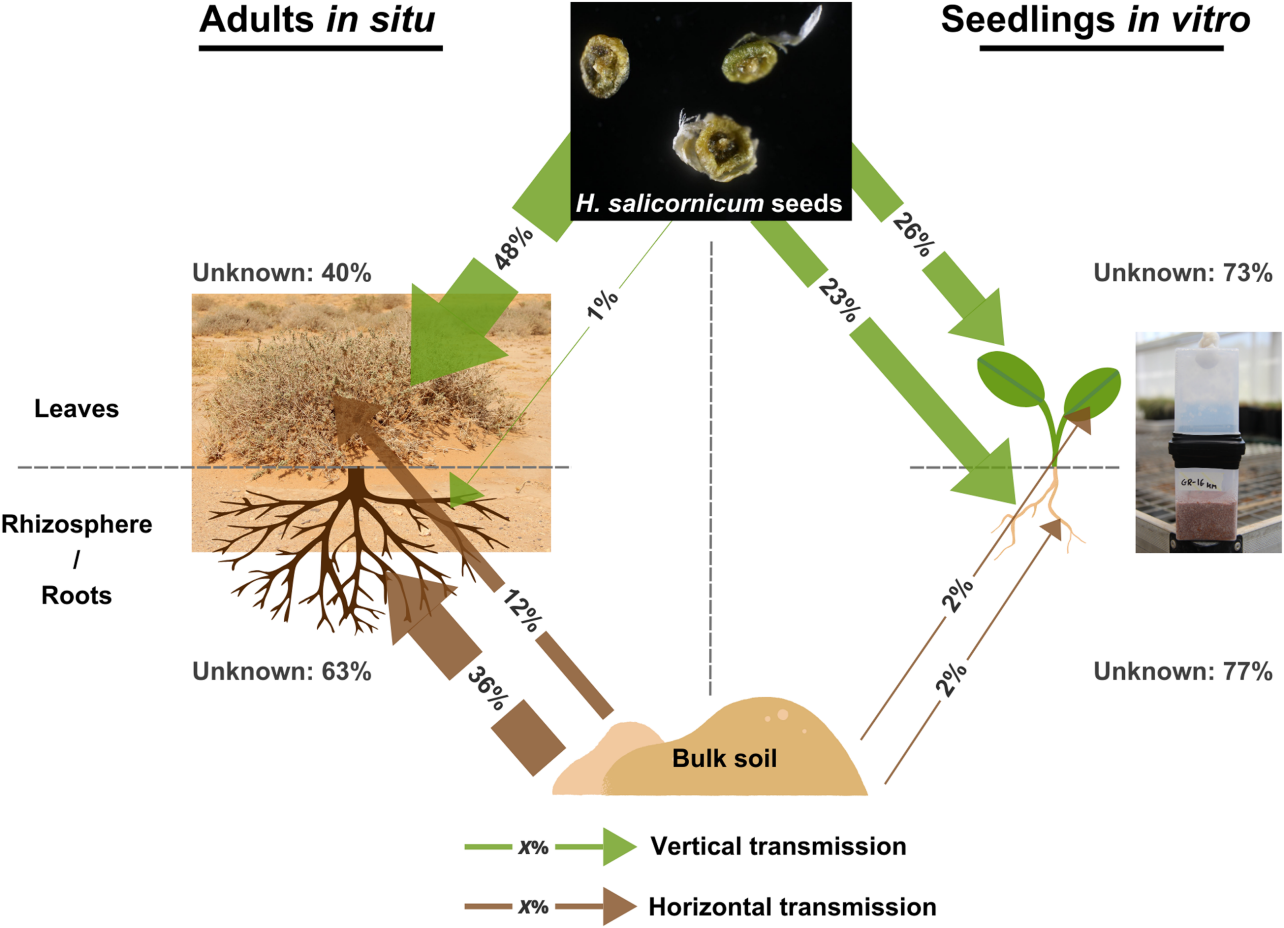
Root and rhizosphere mycobiota of adults in situ are mainly explained by the bulk soil (horizontal transmission), whereas adults' leaf mycobiota in situ and seedlings' leaf and root mycobiota in vitro are mainly explained by seeds (vertical transmission). Graphical summary of the estimated contribution of seeds and bulk soil to *H*. *salicornicum* mycobiota in each compartment using the *FEAST* source tracking algorithm. Vertical transmission (through seeds) explains most of the mycobiota of adults' leaves in situ and seedlings' roots and leaves in vitro. Horizontal transmission (bulk soil) is the main source of roots and rhizosphere mycobiota of adults in situ but also contributes to leaves mycobiota. Arrows width is proportional to the estimated contribution. For readability purposes, the contributions of bulk soil to roots and rhizosphere mycobiota of adults in situ were averaged as they are not significantly different (Figure 5). Similarly, as the contribution of both seeds and bulk soil to leaf and root mycobiota in seedlings in vitro are not significantly different (Figure 5), they were averaged. The unexplained contribution to the mycobiota of adults in situ and seedlings in vitro is also reported.

We comprehensively quantified the respective contribution of vertical (seeds) and horizontal (bulk soil) transmission pathways to *H*. *salicornicum* mycobiota in seedlings in vitro and fully developed individuals in situ, for aerial and underground compartments. It is, to our knowledge, the first time that both transmission pathways were simultaneously quantified at different development stages, in particular in desert ecosystems. We showed that roots and rhizosphere mycobiota of adult individuals in situ are mainly explained by bulk soil (horizontal transmission) with almost no contribution of seeds, whereas roots and leaves of seedlings in vitro are mainly explained by the seeds (vertical transmission) with almost no contribution from the soil (Figure 7). Only leaves of adults in situ display a mixed pattern with a predominance of vertical transmission. Here, transmission from seeds may be pseudo‐vertical as we investigated the total seed mycobiota (epiphyte and endophyte) collected on *H*. *salicornicum* individuals (before falling on the ground) and germinated them without surface sterilization. Vertical transmission sensu stricto describes the transmission of microorganisms strictly to the progeny, that is, with no contamination from the environment (Bright & Bulgheresi, 2010; Truyens et al., 2015). However, seeds are exposed to several environmental contaminations over their life cycle from the early development stage to maturation and germination (Abdelfattah, Tack, Lobato, et al., 2022; Abdelfattah, Tack, Wasserman, et al., 2022; Nelson, 2018), resulting in modified microbiota/mycobiota which are also transmitted to seedlings. Once pseudo‐vertical transmission is ensured, it may allow a partner fidelity sufficient to ensure the evolution of important functions for the host plant (Séne et al., 2018).

### *Vertical transmission is predominant in* H. salicornicum *seedlings' mycobiota in vitro while bulk soil has a quasi‐null influence*

Both leaves and roots of seedlings germinated in vitro in (non‐)autoclaved soil collected in situ display similar mycobiota composition resembling that of seeds, suggesting a predominance of vertical transmission. Due to a low germination rate and events of damping off in previous trials, we collected seedlings in vitro at an early development stage (two‐leaf, 7 days). Despite a limited number of seedlings, we observed strong patterns across samples. First, the mycobiota of seedling roots and leaves in vitro were almost undifferentiated. Yet, compartment is known to be a strong factor of differentiation of the mycobiota, especially between above‐ and belowground compartments (Harrison & Griffin, 2020; Martins et al., 2016; Wearn et al., 2012), even though differences at juvenile stages may be lower as distance between compartments is reduced. Second, both substrate conditions (non‐ and autoclaved) used in the in vitro experiment resulted in similar mycobiota composition after 7 days, suggesting a low influence of this reservoir on seedling mycobiota. As further evidenced by bipartite networks, source tracking analysis, and the identification of potentially transmitted OTUs from seeds to seedlings (leaves and roots), vertical transmission predominates in seedlings mycobiota assembly, contrary to our hypothesis. Our results are at odds with those of Rochefort et al. (2021) as they observed, in *B*. *napus* (Brassicaceae), that differences in the initial quantity of soil microorganisms in the germination substrate led to distinct fungal communities at 7 and 14 days suggesting that soil microbiota has a significantly greater influence on seedling mycobiota than seeds. These differences may be linked to differences in in vitro experimental settings as Rochefort et al. (2021) used gamma‐irradiated soil inoculated with ‘active’ soil suspensions, or differences of mycobiota assembly strategies between species. Other studies have also shown that bacterial microbiota is vertically transmitted to seedlings by seeds (Moroenyane et al., 2021 on soybean; Walsh et al., 2021 on wheat) using inoculated seeds germinated in vitro. In our experiment, we used as substrate soil collected in situ which was unprocessed as much as possible before setting up the in vitro experiment. Though sampling may have altered the physiological condition and colonization capacity of soil fungi by breaking hyphae and limiting access to water, fungi in desert ecosystems have developed adaptations to these harsh conditions (sandy soil, nutrition depletion, low water availability, heat…), such as resistant spores, melanized hyphae, or biofilm formation with other organisms such as bacteria (Ameen et al., 2021; Sterflinger et al., 2012). We therefore hypothesize that soil sampling only had a limited effect on the colonization capacity of soil fungi in our experiment. Furthermore, soil used as substrate in the in vitro experiment was collected after (May 2022) soil samples collected for molecular analysis (March 2022) and may therefore have a different mycobiota due to seasonal variations. In desert ecosystems, though, changes in soil microbiota are primarily linked to rains (Eshel et al., 2021) which did not occur between the two sampling times. In addition, bulk soil samples collected at a 7‐month interval with no rains in different sites of the Sharaan Nature Reserve showed low differences in mycobiota composition (Maurice et al., 2024). These experimental biases could be overcome by sampling seedlings in situ (when possible) in addition to in vitro experiments. A large share of seedling mycobiota in vitro is not explained by seeds or soil (75% in average). Rarefaction curves of both sources (bulk soil and seeds) tend to reach a plateau for most of the samples, suggesting that our sampling properly describes their fungal diversity. Furthermore, we used extraction and PCR negative controls along with stringent filtering to limit the presence of contaminants from molecular analysis. Unexplained fungal transmission may thus occur from other sources of contamination such as air born fungi from the growth room (Zhou et al., 2021), water used for watering of the Magenta boxes, or contaminations from human manipulations. However, our in vitro experimental setup should limit these (Experimental procedure section) as we used closed Magenta boxes to avoid contaminants from the growth room, sterilized water, and tweezers for seeds and sample manipulation. Finally, seedlings were collected and processed under a laminar flow cabinet to avoid contaminations. Despite these precautions, contaminations from sources cited above may have occurred.

Fungi potentially transmitted from seeds to seedlings were similar in the two compartments (roots and leaves) and substrate conditions (non‐ and autoclaved), and were mainly *M*. *asteroma* (ca. 10% of the seedling mycobiota). *Mycosphaerella* spp. have been mainly reported as plant pathogens (Hunter et al., 2011), yet we did not find any visual traces of plant diseases on seedlings at sampling time. Indeed, some *Mycosphaerella* species occur as symptomless endophytes (González‐Teuber, 2016; Kaneko et al., 2003) and shift from pathogenic to endophytic, a behaviour which is common among fungi (Selosse et al., 2018). These endophytic fungi may therefore be either harmless or latent pathogens. Other fungi belong to the yeast genera *Leucosporidium*, the genus *Vishniacozyma*, and *Aureobasidium* (*A*. *pullulans*), which has anti‐fungal activities (Wachowska & Głowacka, 2014). Their roles as seed and seedling endophytes are poorly understood and deserve further attention. As shown in previous studies, the initial mycobiota may impact the recruitment of fungi after germination by either limiting the development of other species or facilitating their establishment in plant tissues, a phenomenon called priority/priming effect (Debray et al., 2022; Ridout et al., 2019). Fungi pseudo‐vertically transmitted may therefore play a crucial role in the mycobiota assembly by facilitating or limiting the colonization of other soil fungi. Such priming effects may be of great importance in desertic ecosystems where (1) harsh soil conditions may limit microbial availability (Maldonado et al., 2022) and (2) large distances between conspecific individuals may limit fungal sharing (Brigham et al., 2023). In particular, seeds may not directly benefit from the higher microbial diversity of fertility islands when dispersed far from *H*. *salicornicum* individuals (Maurice, Laurent‐Webb, et al., 2023). Vertical transmission of fungi in desert ecosystems may thus improve seedlings settling by increasing access to the low water and nutrients (Yakti et al., 2018) and therefore be favoured against horizontal transmission, explaining differences of patterns with other studies (Rochefort et al., 2021). Mycobiota assembly strategies in desert ecosystem, and in particular the high importance of vertical transmission, deserve further attention as they can provide information on plant adaptation to harsh conditions, with potential applications in restauration and desertification mitigation.

### Rhizosphere and root mycobiota of adults in situ are mainly obtained by horizontal transmission, while leaf mycobiota are acquired by both vertical and horizontal transmission

Contrary to seedlings in vitro, adult individuals' in situ display contrasted mycobiota composition according to the compartment studied. The main differences were observed between above‐ and belowground plant compartments (rhizosphere and roots vs. leaves and seeds). These observations are consistent with the source tracking analysis as both rhizosphere and root mycobiota are mainly explained by the bulk soil (39% and 33%, respectively), while the contribution of seeds is quasi‐null. These results suggest that the mycobiota of belowground tissues of adults in situ are mainly transmitted horizontally, as hypothesized in this study. Potentially transmitted OTUs have similar taxonomy and are mainly affiliated to *Penicillium oxalicum* (ca. 10% of the root and rhizosphere mycobiota), *Eutypella* sp. (4%), Pleosporaceae sp. (3.5%), and *Chaetomium* sp. (3%). *P*. *oxalicum* is commonly found in the rhizosphere and was reported as a plant growth‐promoting fungus which could limit the development of some pathogens such as *Fusarium* spp. (Murali & Amruthesh, 2015), while the others may be plant pathogens (esp. *Eutypella* sp.) or endophytes (such as *Chaetomium* sp.; FUNGuild online database, Nguyen et al., 2016).

Leaf mycobiota of adult individuals' in situ display a mixed pattern of colonization. Their composition is similar to seeds which are the main contributor to their mycobiota (48%). This contribution is mainly supported by ubiquitous fungi such as *Alternaria consortialis* which represent a large share of leaf mycobiota in situ (10%) when using the loose definition of potentially transmitted OTUs. As seeds and leaves were collected the same year, we cannot exclude that both compartments influence each other's mycobiota, as confirmed by *FEAST* analyses. Evaluating the contribution of seeds to other compartments on two or more generations would allow to better evaluate the contribution of leaves to the establishment of seeds mycobiota (generation 1) and the contribution of seeds to other compartments (generation 2 and beyond) but this is challenging for perennial plant species with a long‐time span. Notably, bulk soil has a non‐null contribution to leaf mycobiota (12%), also characterized by the contribution of *A*. *consortialis*. Species from the *Alternaria* genus are common seed endophytes (Simonin et al., 2022) and have also been reported as widespread saprotroph and plant pathogens in leaves and shoots (Dang et al., 2015). *Alternaria* spp. have already been isolated from desert soils in the Arabian desert (Ameen et al., 2021) and our results suggest that they may have been recruited from both bulk soil and seeds in situ. Contribution from soil to phyllosphere mycobiota has already been shown for bacteria (Xiong et al., 2021), but the mechanisms of transmission from soil to phyllosphere are poorly described. Some authors suggest that soilborne microbes may be transmitted by air (Zhou et al., 2021) or within plant tissues (Vandenkoornhuyse et al., 2015) and indeed desert soils are poorly covered by vegetation, allowing transfer of dust to air. Our results support transmission of soil fungi to the phyllosphere, but do not allow us to conclude the pathways followed by these fungi. Our results emphasize the importance of considering both vertical and horizontal transmission simultaneously to identify ubiquitous fungi that may be transmitted both vertically and horizontally and therefore avoiding over‐ or underestimation of their respective contribution.

As for seedlings in vitro, a large share of in situ adult mycobiota is not explained by seeds or bulk soil. Contrary to the in vitro experiment, sources of contaminations in situ may be more diverse, including fungi dispersed by wind and small mammals (Borgmann‐Winter et al., 2023; Zhou et al., 2021), or even plant debris from the litter (Christian et al., 2017). Though fungi may be transmitted by wind (e.g., spores), the low density of plant individuals (in particular *H*. *salicornicum* individuals; fertility islands hypothesis) may limit their dispersion and therefore favour the withholding of vertically‐transmitted fungi in *H*. *salicornicum* leaves, while the mycobiota of belowground tissues are more likely to be influenced by the environment (horizontal transmission).

### Vertical and horizontal transmission during microbiota assembly of a desert shrub

Part of the differences in mycobiota composition observed between compartments may also be linked to sequencing bias as samples were sequenced in two different amplicons. Though this bias seems limited for second‐generation sequencing platforms as Illumina MiSeq (Tedersoo et al., 2022), sequencing all samples in one amplicon with platforms yielding a higher number of reads (and thus allowing to sequence more samples at once) such as Illumina NovaSeq could limit potential sequencing bias. Differences in vertical and horizontal transmission observed between seedlings in vitro and adults in situ may be linked not only to differences in life conditions (in situ vs. in vitro) but also to age, and reflect the juvenile mycobiota, which may change during *H*. *salicornicum* development from the establishment of seedlings to adult individuals, as often reported (Gao et al., 2019; Han et al., 2017; Houlden et al., 2008; Liu & Howell, 2021). In the latter case, our results suggest a secondary colonization of roots and rhizosphere by soil fungi, replacing fungi vertically transmitted to seedlings. Plants can indeed recruit fungal partners actively by emitting signal molecules (Daguerre et al., 2020), while some fungi colonize compartments without any active recruitment from the plant (Gao et al., 2020), leading to changes in mycobiota composition with age (ecological recruitment process). Differences in horizontal and vertical transmission between in situ adults and in vitro seedlings are less contrasted in leaf mycobiota as in both cases, seeds (vertical transmission) are the main contributor to their mycobiota. However, we identified a significant share of horizontal transmission from the soil to leaves of adults in situ but none in seedlings in vitro. Furthermore, fungi potentially transmitted from seeds to leaves differ between adults in situ and seedlings in vitro. Again, these differences may be linked to differences between environmental conditions, but also to changes in mycobiota composition of aerial compartments during plant development (Maignien et al., 2014).

Finally, understanding whether fungi are transmitted horizontally or vertically has crucial ecological implications over short timescales, but may also help us to understand phenomena occurring over long timescales such as phylosymbiosis patterns (i.e., phylogenetically close individuals tend to associate preferentially with related hosts), which have been observed in plants with fungi (Perez‐Lamarque et al., 2020) and bacteria (Abdelfattah, Tack, Lobato, et al., 2022; Abdelfattah, Tack, Wasserman, et al., 2022), interactions suggesting preferential associations between plants and fungi and/or bacteria. Some authors suggest that such evolutionary patterns are testimony of microbiota transmission from one generation to the other (Abdelfattah, Tack, Lobato, et al., 2022; Abdelfattah, Tack, Wasserman, et al., 2022), reflecting the relevance of our findings, although host filtering from the environment may be the main mechanism at stake (Mazel et al., 2018).

## CONCLUSION

Through a combination of in situ sampling and in vitro experiment, we have comprehensively described and quantified vertical (from seeds) and horizontal transmission (from bulk soil) of *H*. *salicornicum* mycobiota (Figure 7). We show in vitro that the mycobiota are partially transmitted to seedlings by seeds (vertical transmission) while soil does not influence their mycobiota, contrary to our hypothesis and previous results. The mycobiota of belowground compartments of in situ adult individuals display an opposite pattern as the contribution of seeds is almost null: rhizosphere and root mycobiota are mainly explained by the bulk soil (horizontal transmission). Leaves of adult individuals display a mixed pattern as their mycobiota are mainly explained by seeds, with a significant contribution of bulk soil. However, fungi transmitted from seeds to leaves differ between adults and seedlings. Differences in transmission pathways between adults in situ and seedlings in vitro may be linked to differences in experimental designs, but also to differences between developmental stages. Taking dynamic changes in the mycobiota and the transmission pathways into account would improve our understanding of mycobiota assembly. Finally, we show that some fungi are specifically shared between a source and a compartment (e.g., bulk soil and roots sharing *P*. *oxalicum*), while some ubiquitous fungi (such as *A*. *consortialis*) may be transmitted by both seeds and soil. The role of these fungi needs further attention, in particular, to decipher whether fungi transmitted by seeds are beneficial or deleterious for the host. A better understanding of the mycobiota assembly processes under strong environmental constrains will be of great importance for natural environment protection and restauration in hot deserts but also to develop new strategies to tackle drought stress in crops or natural environments facing desertification.

## AUTHOR CONTRIBUTIONS

**Liam Laurent‐Webb:** Conceptualization (equal); formal analysis (lead); investigation (equal); methodology (equal); resources (equal); visualization (lead); writing—original draft (lead); writing—review and editing (equal). **Kenji Maurice:** Conceptualization (equal); data curation (equal); investigation (equal); resources (equal); validation (equal); writing—review and editing (equal). **Benoît Perez‐Lamarque:** Formal analysis (equal); methodology (equal); writing—review and editing (equal). **Amélia Bourceret:** Investigation (equal); project administration (equal); supervision (equal); writing—review and editing (equal). **Marc Ducousso:** Conceptualization (equal); funding acquisition (lead); project administration (equal); supervision (equal). **Marc‐André Selosse:** Conceptualization (equal); funding acquisition (lead); project administration (equal); supervision (equal).

## CONFLICT OF INTEREST STATEMENT

The authors declare no conflicts of interest.

## Supporting information


**Data S1:** Supporting Information.

**File S1:** Supplementary File.

**File S2:** Supplementary File.

## Data Availability

Sequencing data are publicly available in GenBank (accession number: PRJNA1028866 for bulk soil, rhizosphere, and roots in situ and PRJNA1035432 for leaves, seeds in situ, and seedling's leaves and roots). The bioinformatics pipeline can be found at: https://github.com/BPerezLamarque/Scripts.
